# Supplementary material for: A Novel Long Non-Coding RNA-01488 Suppressed Metastasis and Tumorigenesis by Inducing miRNAs That Reduce Vimentin Expression and Ubiquitination of Cyclin E
Source: Cells. 2020 Jun 20;9(6):1504. doi: 10.3390/cells9061504 (PMC7348830; doi:10.3390/cells9061504)
Supplement: Supplementary file 1 [file cells-09-01504-s001.pdf]

Supplementary materials:

Figure S1. The list of top10 dysregulated lincRNAs in two HCCs by microarray analysis.

Figure S1.

Downregulation or upregulation of lincRNA profiling from two HCC samples (Top 1-10)

Down regulation of lincRNA	Up regulation of lincRNA
LOC255167	AGSK1
LOC100506229	ANKRD20A8P
C3P1	ANXA2P1
XLOC_003629	ANXA2P3
FAM99A	ARGFXP2
LOC100507389	AURKAPS1
XLOC_009194 (LINC01488)	C12orf32
XLOC_l2_013064	C12orf47
LOC100271831	C17orf76-AS1
XLOC_l2_011612	C17orf88

Figure S2. The EMT markers in LINC01488 depleted- or overexpressing-hepatoma stable cells determined via immunofluorescence assay.

Figure S2.

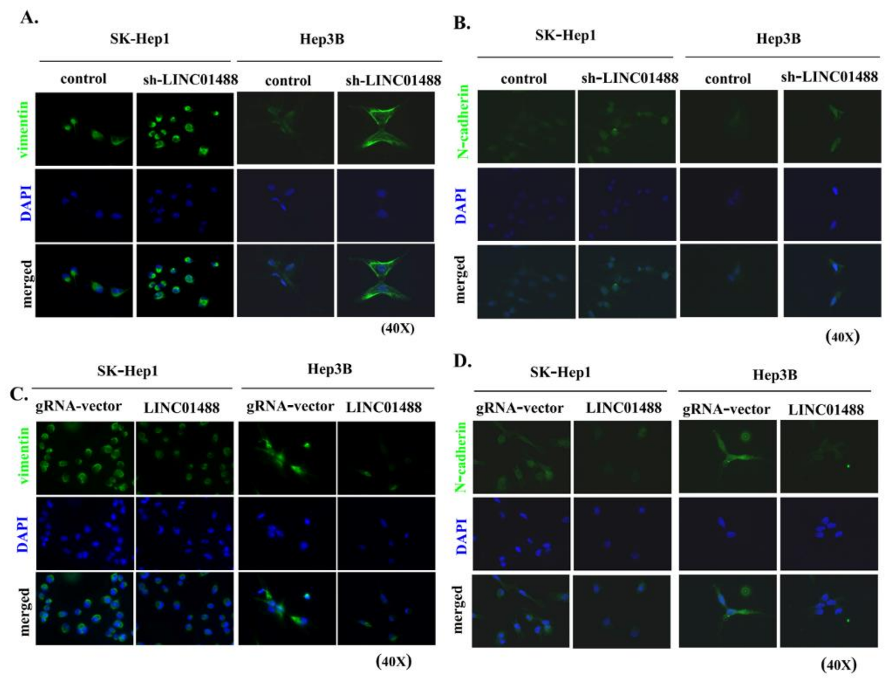

(A, B) Depletion of LINC01488 affected EMT expression levels in SK-Hep1 or Hep3B cells. (C, D) Forced expression of LINC01488 suppressed EMT expression levels in SK-Hep1 or Hep3B cells.

Figure S3. LINC01488 regulates the anti-metastatic/anti-proliferative mechanism through vimentin/cyclin E, respectively.

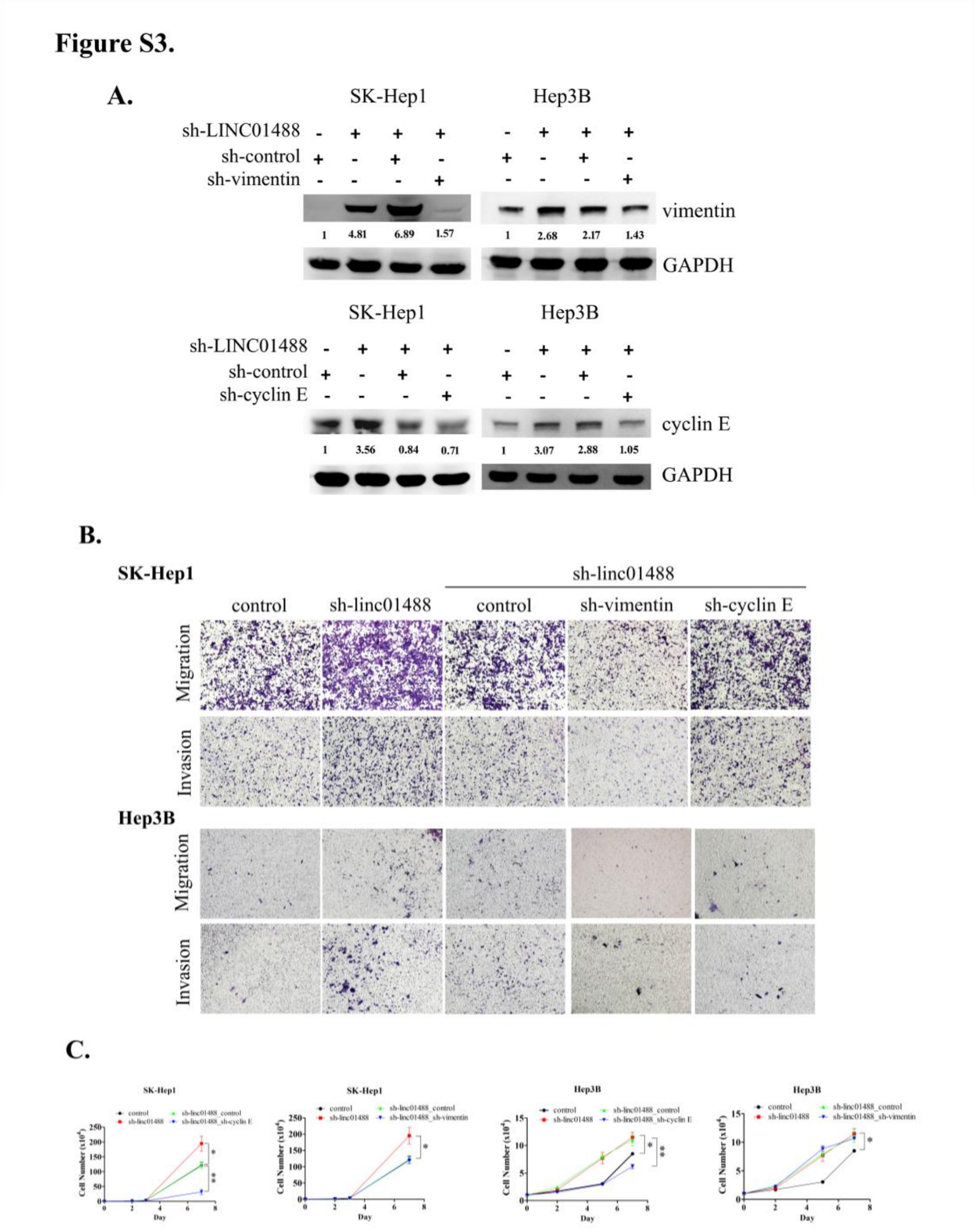

(A) The protein expression levels of vimentin- or cyclin E-knockdown in LINC01488-depleted cells (SK-Hep1 and Hep3B) were assayed by western blot. (B) Further, the migratory/invasive capabilities were determined in vimentin-knockdown as well as LINC01488-depleted cells compared with ctrl-shRNA cells (SK-Hep1 and Hep3B). (C) In proliferative assay, cyclin E-shRNA (blue) or vimentin-shRNA(blue) knockdown in LINC01488-depledted cells was determined compared with the ctrl-shRNA (green) (SK-Hep1 and Hep3B). Data are presented as means ± SD (*p<0.05; **p<0.01).

Figure S4. LINC01488 don't bind to vimentin via RIP assay.

Figure S4.

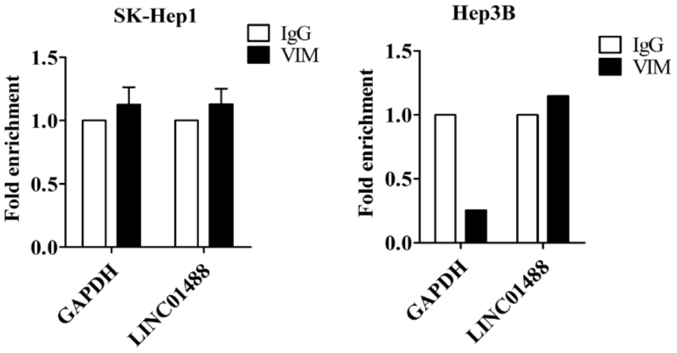

RNA immunoprecipitation (RIP) assay to determine interactions between LINC01488 and vimentin in SK-Hep1 and Hep3B cells. Data are presented as means ± SD (*p<0.05; **p<0.01).

Figure S5. LINC01488 doesn't interact with CDKs by RIP assay.

Figure S5.

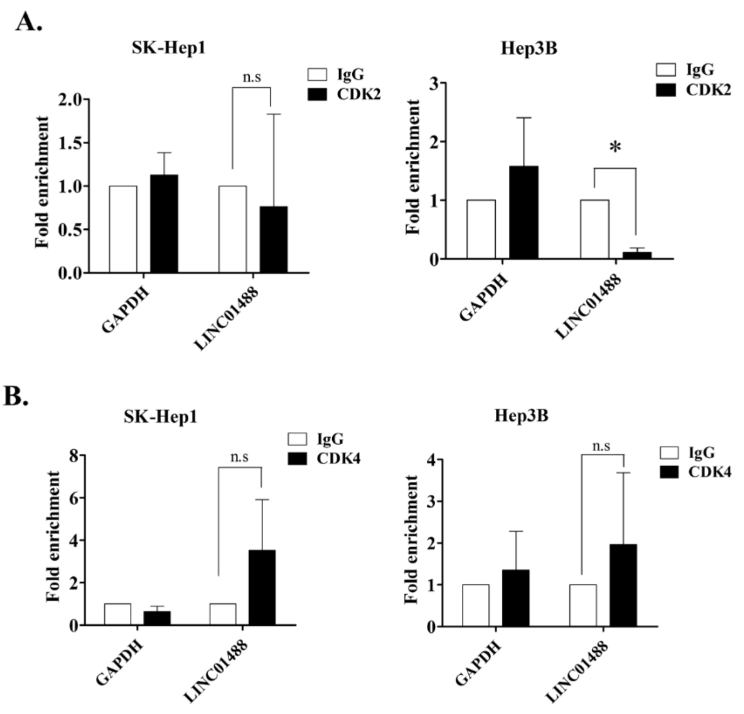

(A, B) RIP assay to determine interactions between LINC01488 and CDK2 or CDK4 in SK-Hep1 or Hep3B cells. Data are presented as means ± SD (*p<0.05; **p<0.01).

Figure S6. The mRNA level of cyclin E don't regulate by LINC01488.

Figure S6.

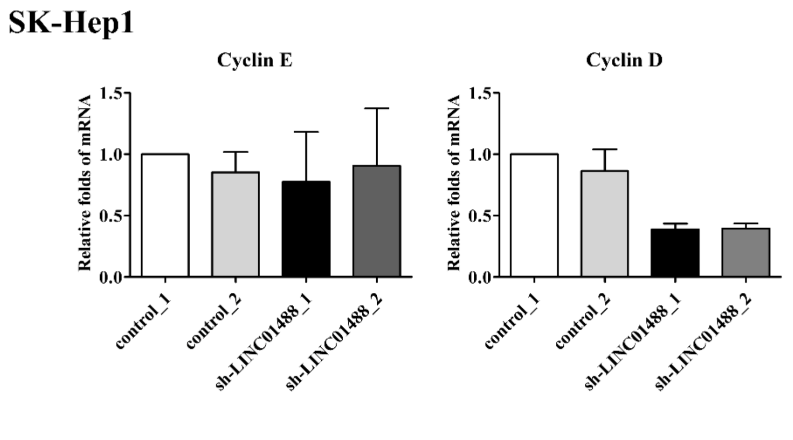

Depletion of LINC01488 don't affect cyclin E and cyclin D expression levels in SK-

Hep1cells, as determined via q-RT-PCR. Data are presented as means ± SD (*p<0.05; **p<0.01).

Figure S7 Lists of primer and sgRNA sequence.

Figure S7

A.

shRNA	Primer sequence
sh_LINC01488#1F	ccggAGCAGGGACACAGCATGAAGGctcgagCCTTCATGCTGTGTCCCTGCTttttt
sh_LINC01488#1R	aattaaaaaAGCAGGGACACAGCATGAAGGctcgagCCTTCATGCTGTGTCCCTGCT
sh_LINC01488#2F	ccggAGCATCATCACAAGGGTGCTTctcgagAAGCACCCTTGTGATGATGCTttttt
sh_LINC01488#1R	aattaaaaaAGCATCATCACAAGGGTGCTTctcgagAAGCACCCTTGTGATGATGCT

B.

Gene	Q-PCR Primer sequence (Forward)	Q-PCR Primer sequence (Reverse)
LINC01488	TTTTGTGTCTGCACTATTTCCAAAG	GGCTGATCTGATGCAGATCCTT
Vimentin	GACGCCATCAACACCGAGTT	CTTTGTCGTTGGTTAGCTGGT
N-cadherin	AGCCAACCTTAACTGAGGAGT	GGCAAGTTGATTGGAGGGATG
MMP-9	GGGACGCAGACATCGTCATC	TCGTCATCGTCGAAATGGGC
Cyclin E	ACTCAACGTGCAAGCCTCG	GCTCAAGAAAGTGCTGATCCC
Cyclin D	CAATGACCCCGCACGATTTC	CATGGAGGGCGGATTGGAA

C.

Sample	sgRNA sequence	score
LINC01488-sgRNA	TGGTGCTGTGGACGGCTTCA**GGG**	80

D.

MicroRNA	Precursor-miR Q-PCR primer (Forward)	Precursor-miR Q-PCR primer (Reverse)
Pre-mir124	AGGCCTCTCTCTCCGTGTTC	CAGCCCCATTCTTGGCATTC
Pre-mir138	CGTTGCTGCAGCTGGTGTTG	TGATGCAACCCTGGTGTCGT

The list of primer sequences for (A) shRNAs of LINC01488, (B) genes of LINC01488, vimentin, N-cadherin, MMP-9, cyclinE and cyclinD, (C) sgRNA of LINC01488 and (D) pre-miRs (miR-124 and miR-138) by qRT-PCR assay.
